# A Fast Decision Algorithm for VVC Intra-Coding Based on Texture Feature and Machine Learning

**DOI:** 10.1155/2022/7675749

**Published:** 2022-09-13

**Authors:** Jinchao Zhao, Peng Li, Qiuwen Zhang

**Affiliations:** College of Computer and Communication Engineering, Zhengzhou University of Light Industry, Zhengzhou 450002, China

## Abstract

Due to the development and application of information technology, a series of modern information technologies represented by 5G, big data, and artificial intelligence are changing rapidly, and people's requirements for video coding standards have become higher. In the High-Efficiency Video Coding (HEVC) standard, the coding block division is not flexible enough, and the prediction mode is not detailed enough. A new generation of Versatile Video Coding (VVC) standards was born. VVC inherits the hybrid coding framework adopted by HEVC, improves the original technology of each module, introduces a series of new coding technologies, and builds on this greatly improving the coding efficiency. Compared with HEVC, the block division structure of VVC has undergone great changes, retaining the quad-tree (QT) division method and increasing the multi-type tree (MTT) division method, which brings high coding complexity. To reduce the computational complexity of VVC coding block division, a fast decision algorithm for VVC intra-frame coding based on texture characteristics and machine learning is proposed. First, we analyze the characteristics of the CU partition structure decision and then use the texture complexity of the CU partition structure decision to terminate the CU partition process early; for CUs that do not meet the early termination of the partition, use the global sample information, local sample information, and context information. The three-category feature-trained tandem classifier framework predicts the division type of CU. The experimental results show that in the full intra mode, compared with the existing VTM10.0, the encoding output bit rate is increased by 1.36%, and the encoding time is saved by 52.63%.

## 1. Introduction

With the advent of the 5G era, new video formats such as short videos, live videos, 4K/8K ultra-high-resolution videos, and virtual reality videos will emerge one after another. Video is changing people's lives in learning, entertainment, and socializing. In the field of mobile communications where bandwidth resources are extremely valuable, Ericsson's latest research report [1] shows that by 2026, video traffic will account for 77% of all mobile data traffic, which will make it impossible to meet the concurrent video transmission needs of a large number of users. The coding performance of video encoders is very important for network transmission and user experience. Currently, videos on the Internet are mainly coded using AVC/H.264 and HEVC/H.265 [2], and there is a large gap between coding performance and practical needs. Therefore, the latest Versatile Video Coding (VVC) standard came into being, which was researched and released by the Joint Video Experts Group (JEVT) formed by VCEG and MPEG. VVC introduces a large number of new encoding tools to improve encoding performance, and the video encoding efficiency is increased by nearly 40% while maintaining the same reconstruction quality. It should be pointed out that the improvement of VVC comes at the cost of increasing coding complexity. Compared with HEVC, the coding time of VVC is increased by about 25 times under the All Intra (AI) configuration, and the encoding time of VVC is increased by about 7 times under the random access (Random Access, RA) configuration [3]. Although the VVC coding structure is very similar to its predecessor HEVC, many new technologies have been added on this basis: QTMT block partition structure, 67 intra-frame prediction modes, intra-frame cross-component linear (CCLM) prediction, adaptive multi-core transform (AMT) technology, matrix weighted intra prediction technology (MIP), motion vector derivation FRUC technology based on pattern matching, optical flow–based bidirectional motion (BIO) compensation, affine (Affine) motion prediction technology, and SubPU-level motion vector prediction technology, relying on scalar quantization technique (DSQP), low-frequency nonseparable transform technique (LFNST), intra-frame subdivision (ISP), etc. [4, 5].

Compared with the macroblock division in HEVC/H.265, the newly added quadtree nested multi-type tree division in VVC makes the coding block size more flexible and more suitable for video coding content. In recent years, many algorithms have emerged to speed up the CU partitioning process or terminate the CU early. It is found that the recursive process of encoding block quadtree nested multi-type tree division occupies most of the encoding time. To reduce the complexity of VVC intra-frame coding, researchers have made a lot of effort. Given the above situation, we propose a fast CU partitioning algorithm. The main contributions of this work are: first, based on the texture characteristics of CU, the characteristics of the CU partition structure decision are analyzed by encoding and decoding the image, and then the CU partition process is terminated early by using the characteristics of the CU partition structure decision. Second, we perform statistical analysis on some key information in the CU partitioning process. Then, a reasonable acceleration strategy is designed according to the analysis results, and representative features are selected. For CUs that do not meet the early termination of the split, train the tandem classifier framework to predict the split type of the CU.

The rest of the specific structure of this paper is arranged as follows. The second section introduces the partition structure of VVC and some related fast algorithms.  Section 3 firstly analyzes the complexity of CU texture, and introduces a fast partition algorithm based on image texture characteristics and a fast partition algorithm based on the tandem classifier framework.  Section 4 presents related experiments and experimental results and compares them with state-of-the-art solutions. Finally, Section 5 summarizes the full text.

## 2. Background and Related Work

### 2.1. Introduction to the QTMT Partition of VVC

The new-generation Versatile Video Coding (VVC) standard has greatly changed its division method based on HEVC. One of the key features of HEVC is the concept of multiple partition units, including the coding unit, the basic unit of the encoding process, the prediction unit (PU), which performs prediction operations, and the transform unit (TU), which performs transform and quantization operations. In HEVC, to be suitable for a variety of different video image textures, a CTU is divided into CUs through a quad-tree (QT) division, and each sub-CU is divided into quad-trees or not divided based on the division selection of the PU. In a child PU, a prediction process similar to that of the parent PU will be performed, and then the decoder will accept the information related to the PU, and then subtract the prediction block obtained by PU division from the original block to obtain the residual block. The parent CU is divided into TUs using another quad-tree structure with the same division structure as the CU, and then the TUs are taken for transform and quantization operations.

**Figure 1:**
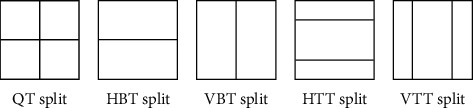
QTMT division type.

Different from HEVC, the difference between CU, PU, and TU are removed in VVC, that is, CU is used for prediction, transformation, quantization, and entropy coding, and it is no longer further divided into PU and TU based on CU. VVC adopts a new coding block division method. Based on quad-tree (QT) division, a multi-type tree (MTT) division method is added. Among them are horizontal binary tree division (HBT), vertical binary tree division (VBT), horizontal Ternary tree division (HTT), and vertical ternary tree division (VTT); the division structure is shown in Figure 1. The division method of quadtree nested multi-type tree in VVC significantly improves the coding performance, which makes the division method more flexible, and is no longer limited to square, but a new rectangular block is added.

**Figure 2:**
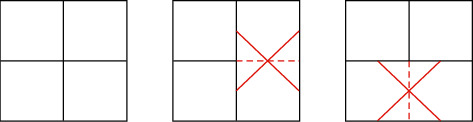
Redundant partition of quadtree partition and binary tree partition.

In VVC, due to some parameter limitations in the encoder, usually, we set the size of a coding tree unit (CTU) to 128 × 128, which must be divided into QT first. The minimum quadtree size (MinQtSize) is set to 8, which means that the minimum allowable size of the QT child node is 8 × 8, and the CU that has been divided into 8 × 8 can only be divided into MT. The maximum binary tree size (MaxBtSize) and the maximum ternary tree size (MaxTtSize) are both 32, which means that the largest MT root node is 32 × 32, that is, the earliest 32 × 32 CU can be divided into binary and ternary trees. The QTMT block division structure makes the division modes of VVC/H.266 various, and different division modes may obtain the same division structure, which results in the appearance of redundant division. For example, as shown in Figure 2, when a coding block is divided by QT, four identical sub-CUs will be obtained, and the same coding block will be divided by BT twice to obtain the same division result. To avoid the occurrence of this redundant situation, we usually impose certain restrictions on the division scheme of the coding block. For example, when a coding block allows QT division, it is not allowed to perform two consecutive BT divisions; and when a coding block does not allow QT division, it can perform two consecutive BT divisions. Two consecutive BT divisions are allowed in the following two cases: First, if the block size of the current quad-leaf node is equal to the minimum length allowed by quad-tree division, it is forbidden to continue to use quad-tree division for the current leaf node, but multiple types of tree partitioning are allowed. Second, if the multi-type tree division depth of the current leaf node to be divided is greater than 0 (the leaf node of the multi-type tree MT), it is forbidden to use quadtree division for the current node [6].

**Figure 3:**
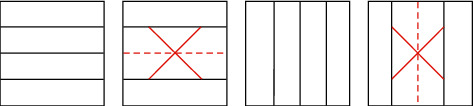
Redundant partition of binary tree partition and ternary tree partition.

As shown in Figure 3, different combinations of binary tree division and ternary tree division are used for the same coding block, and the same coding block structure may appear. The coding block structure obtained after the coding block is divided into two BT divisions in the same direction is the same as the coding block structure obtained by first TT division and then the BT division of the central block. Therefore, the general video coding standard prohibits the use of BT partitioning in the corresponding direction in the central partition of the ternary tree.

**Figure 4:**
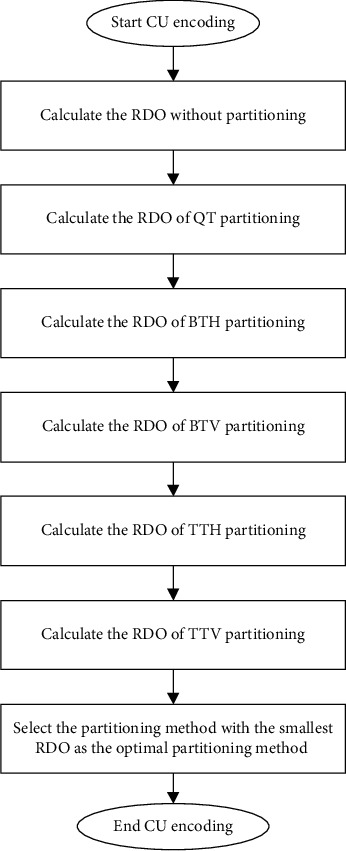
CU optimal partition mode selection process.

In VVC/H.266, the main content of video coding lies in the coding block division, and the flexible block division structure achieves good coding performance. VVC/H.266 adopts a more flexible division structure, using quad-tree nested multi-type tree division to adapt to texture features in video sequences. To find the best combination of the CTU partition mode and the corresponding intra mode, firstly, it is divided into four square sub-CUs of equal size by the quadtree (QT) structure, and then, the sub-CUs can be divided by the quadtree (QT) structure. The CU is recursively divided into square CUs, or the sub-CUs are divided into rectangular CUs by multi-type tree (MT) structure division, and finally, the VTM traverses all possible partition combinations to perform a rate-distortion optimization (RDO) process, finding each CTU the optimal partition structure of the corresponding intra prediction mode, and the one with the lowest rate-distortion optimization (RDO) cost is selected as the best mode. The CU division of H.266/VVC is a recursive process, as shown in Figure 4.

**Figure 5:**
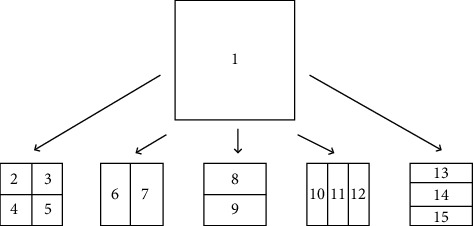
The mode of division of square CU in VVC.

In intra prediction, the coding block recursively tries all possible partition combinations until the best CU partition decision is made, and the partition structure with the least rate-distortion cost is selected from it. Although this division method can obtain the globally optimal partition structure, it also consumes a lot of time. In HEVC/H.265, under the same depth, a square coding block has 5 modes for partition determination. However, in VVC/H.266, a square coding block has 15 modes for partition determination, as shown in Figure 5.

### 2.2. Related Work

The key to video coding research is to find redundancies in video signals and develop compression tools using these redundancies. Compared with HEVC, VVC has undergone great changes. Whether it is the change of the coding block division mode, the increase of the intra-frame mode, the improvement of motion estimation, etc., the video compression performance has been significantly improved, but it also brings complexity degree increase. To minimize the time used for encoding while ensuring performance, it is necessary to propose an effective improvement method to change this status quo.

#### 2.2.1. Research Status of HEVC Express Coding

There are many methods for accelerating CU partition in HEVC/H.265 video coding, which can be divided into three categories: traditional heuristic, deep learning, and neural network based.

*(1) Traditional Heuristic Methods*. In recent years, some fast CU partition algorithms based on heuristic methods have been frequently adopted, and representative methods include Refs. [7–12]. Shang et al. [7] used the depth information of adjacent CUs of coding blocks for early termination decision of CU partition or CU pruning. Kim et al. [8] made a statistical analysis of the rate-distortion cost in the process of coding block division and proposed an algorithm for early termination of CU division. Mallikarachchi et al. [9] counted and analyzed the local range (LR) value and its variance of pixels in the neighborhood to determine the texture uniformity of the coding block, and proposed a fast CU partition decision algorithm based on local range characteristics. Shen et al. [10] proposed a fast CU partition decision algorithm based on texture attributes and depth information of adjacent encoded CUs. The texture features of the CU are first used to select the early determination of the CU size decision, and the spatial correlation of the coding units is used to terminate the CU partition early. Min et al. [11] extracted the local and global edge complexity in the horizontal, vertical, 45° diagonal, and 135° diagonal directions of the coding block, and determined the CU partition mode according to the set threshold. Zhang et al. [12] compared the information at the micro and macro levels of each coding unit block and proposed a fast CU size decision algorithm based on image complexity by applying adaptive processing to entropy and texture contrast.

*(2) Machine Learning Methods*. Machine learning due to its excellent performance, researchers began to introduce machine learning into the field of video compression, and representative methods include Refs. [13–18]. Mu et al. [13] designed a coding block acceleration algorithm based on a support vector machine (SVM). This SVM carefully studies the relationship between rate-distortion (R-D) cost and CU depth using mean square error (MSE) and several encoded bits (NEB) metrics. Zhang et al. [14] designed two SVMs to perform CU's partition mode decision and early termination decision, respectively. The selected features include three categories: the Hadamard cost of the current CU, the depth difference between the current CU and neighboring CUs, and the rate-distortion cost of the current CU and neighboring CUs. Liu et al. [15] proposed an adaptive fast CU size decision algorithm based on SVM. The SVM uses texture complexity as a feature for training, and the output is the complexity level of the CU, including high, low, and uncertain, corresponding to three division decisions: division, no division, and uncertainty, respectively. Zhu et al. [16] designed a binary multivariate classification support vector machine (SVM), which designed the decision function of SVM to achieve a balance between RD performance and coding complexity. Wang [17] et al. obtained dynamic partition parameters of coding tree units through local features and designed a joint decision tree framework to terminate the CU partition early. A fast-coding unit (CU) partition decision method based on a decision tree classifier by Gellert et al. [18] selects and applies a decision tree model by analyzing new coding features related to CU partition decisions.

*(3) Deep Learning Methods*. Compared with machine learning methods, convolutional neural networks (CNN) do not need to consider what features to use due to their characteristics, and are more and more popular among video coding researchers. Some researchers use the trained neural network to predict the CU partition mode to reduce the high computational complexity, and representative methods include Refs. [19–23]. Liu et al. [19] constructed a shallow Convolutional Neural Network (CNN) to predict the partition pattern of CU. Feng et al. [20] proposed a CNN-based CU depth range prediction algorithm to predict the depth range of CU. Zhang et al. [21] combined heuristic methods with deep learning methods. First, a threshold-based texture classification is performed to terminate the partitioning process of the flat-type CU. Then, three different CNNs are designed to predict the partition patterns of nonflat types of CUs. Chen et al. [22] designed a convolutional neural network with an asymmetric kernel, which can more accurately extract texture features from cross-purple, and predict the division of CU. Xu et al. [23] designed a hierarchical neural network framework with early termination, which can output the division results of the entire encoding block, thus significantly reducing the encoding complexity.

#### 2.2.2. Research Status of VVC Express Coding

All the above methods are researched for HEVC standards, which can greatly reduce the complexity of HEVC encoding. Compared with HEVC, the main change of VVC is the change in the coding block structure. The QTMT structure introduced by it can greatly reduce the coding bit rate, but its high complexity limits its application. Therefore, the research on the QTMT structure and the corresponding intra-frame fast decision-making algorithm have appeared, as follows.

Cui et al. [24] determined the probability of each division mode by analyzing and calculating the gradient ratio and threshold in the horizontal and vertical directions and skipped the division mode with a lower probability. Fan et al. [25] proposed a fast CU partition decision algorithm based on variance and gradient. First, it is judged whether to further divide the CU according to the texture complexity. Then, use the gradient to determine whether to partition the CU by the quadtree. Finally, an optimal partition model is selected according to the complexity difference of the sub-CUs. Yang et al. [26] designed a fast algorithm for CU partitioning based on the joint framework of sub-consecutive decision trees. First, the depth distribution of CUs is statistically analyzed. Then, a joint classification framework is designed to output the probabilities of various divisions. Finally, a division mode with a probability greater than 90% is selected as a candidate for the final division mode. Zhang et al. [27] proposed a random forest-based coding block partition decision algorithm. First, the texture complexity of the encoded block is calculated, which is divided into three types: complex, simple, and normal. Then, a random forest classifier is used to output a partition pattern that selects complex types of CUs. Finally, the partitioning process of the simple type CU is terminated. Abdallah et al. [28] designed an early terminated CNN network to predict the 64 × 64 coding block quadtree division structure, and finally obtained a 128 × 128 coding tree division structure. Tissier et al. [29] divided a 64 × 64 CU into 256 4 × 4 CUs and used CNN to predict the probability that the boundaries of each 4 × 4 CU existed. Abdallah et al. [30] proposed a fast CU partition algorithm based on CNN and designed a neural network structure named CNN-BTH to predict the decision depth of 32 × 32 CU horizontal binary tree division. Chen et al. [31] used the pixel variance value of the original image to terminate the further division of the CU with a size of 32 × 32 in advance. If the conditions for early termination were not met, the Sobel operator was used to extract the gradient features of the CU and calculate the sub-CU's variance, selecting the most probable division from five divisions.

The above acceleration algorithms have a good effect on reducing the complexity of video coding. Although they have certain reference values for our research, they all have certain shortcomings. For example, in the literature [24] based on traditional methods, its prediction accuracy is not high. Literature [25] based on variance and gradient methods, although it achieves good results in encoding time savings, it achieves high encoding loss. Based on the deep learning method [28], it can only predict the QT partition structure of 64 × 64 coded blocks but cannot predict the maximum depth of each layer. These methods are very effective and innovative, but there is still room for improvement in coding efficiency. These existing works can greatly reduce the complexity of VVC intra-coding, but the different trade-offs between complexity reduction and coding loss have not been investigated. According to the research status of the above acceleration algorithms, we aim at the complex coding structure used in H.266/VVC video coding, to save coding time, hardly increase the coding bit rate, and ensure good decoded video quality. Therefore, we study the fast decision algorithm of H.266/VVC intra-frame coding.

## 3. Methodology

### 3.1. CU Texture Complexity Detection and CU Partitioning

In the literature [32], we can find that if the block division skips the binary tree division, it can reduce the coding time by 75% on average, and skipping the ternary tree division can reduce the coding time by 48%. If the multi-type tree division is skipped, the encoding time can be reduced by an average of 92%. During the CU partitioning process, some algorithms can be used to quickly find the optimal partition structure of the CU. In the process of intra-frame prediction, we usually divide the area with complex textures multiple times. To obtain the optimal partition architecture of CU, the area with relatively simple texture and homogeneity is regarded as a whole [24]. Areas with complex textures, to ensure the efficiency of video coding, need to be further divided into smaller coding blocks. If viewed as a whole, the partition structure of the coding blocks cannot be accurately predicted. For areas with simple texture, only one intra-prediction mode is used, that is, the pixel value of the coded block can be accurately predicted. If the depth division of the coding block can be terminated in advance or the low-probability division method can be skipped, the computational complexity of the CU division will be effectively reduced on the premise of ensuring coding efficiency and image quality. Simple textured, flat areas in an image are usually encoded as a whole. Therefore, for areas with simple and flat textures in the image, early termination of depth can be performed to reduce the computational complexity of the intra-prediction mode.

**Figure 6:**
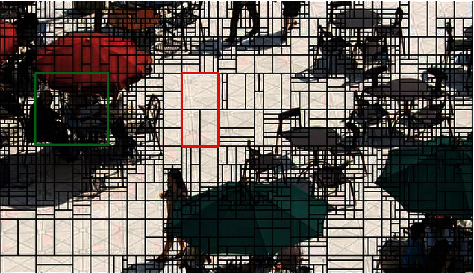
The image after BQSquare encoding and decoding.

To verify the relationship between texture and CU division, this paper selects the test sequence “BQSquare” for encoding and decoding processing.  Figure 6 shows the encoded and decoded image of the test sequence “BQSquare.” It can be seen from the image that the area with simple or homogeneous texture, such as the red border area of the image, tends to be divided into a whole for coding, so the size of the coding block is relatively large; for areas with complex textures, such as the green border area of the image, the size of the coding block is usually smaller, which can better predict the internal structure of the image.

From the analysis of Figure 6, an important conclusion can be drawn: the complexity of the division structure of CU is almost the same as the complexity of texture; usually, the area with simple or flat texture is divided into a whole, and the probability of division is very small; the higher is the probability of CU division in areas with complex textures. Based on this conclusion, to measure the texture complexity, we use the mean absolute difference to represent the overall texture complexity of the CU, as shown in the following equation:

(1) $$\begin{array}{c} MAD=\frac{1}{width\times height}\overset{width}{\underset{i=1}{\sum }}\overset{height}{\underset{j=1}{\sum }}\left|P\left(i,j\right)-Mean\right|, \end{array}$$

where width and height represent the width and height of the coding block, respectively, *P*(*i*, *j*) is the pixel value at (*i*, *j*), and mean is the average value of the pixels in the coding block. To better represent the discrete degree of data, we calculate the mean value of the absolute difference of all pixel values in CU and CU pixels and then compare the mean value of the absolute difference between CU pixels with the mean value of pixels. If the ratio of the absolute difference means to the pixel mean is less than the threshold T, the current coding block is defined as an area with simple texture. If the ratio of the absolute difference means to the pixel mean is less than the threshold *T*, then define the current coded block as a texture-simple region.

**Figure 7:**
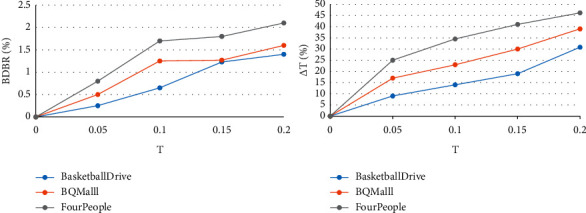
The relationship between threshold *T* and BDBR, △*T*.

To obtain the most suitable threshold *T*, we selected some videos from the test sequence for testing. One test sequence was selected from each of Class B, Class C, and Class E video sequences. These test sequences are “BasketballDrive,” “BQMalll,” and “FourPeople.” The video sequences selected for this test are representative to a certain extent. The relationship curve between the threshold *T* and its corresponding coding efficiency loss and coding time saving is shown in Figure 7. The abscissa in the figure represents the threshold, and the ordinate represents the coding efficiency loss and coding time saving, respectively. The corresponding coding efficiency loss after video coding is represented by the coding bit rate (BDBR). BDBR represents the bit rate that the comparison algorithm can save under the premise of ensuring the same objective quality of the image. Coding time-saving △*T* represents the percentage of coding time saved by comparing algorithms under the same test conditions.

Considering the balance between coding efficiency and coding time, we finally decided to use 0.05 as the threshold. If the given threshold conditions are met, it means that the texture of the current CU is relatively simple and does not need to be further divided, and the CU depth division can be terminated early.

### 3.2. The Proposed Tandem Classifier Framework

According to our analysis of CU texture features, the computational complexity of coding can be reduced to a certain extent by early termination of depth division for CUs with simple textures. However, for areas with complex textures and texture edges, this CU fast partition algorithm cannot be applied to reduce the computational complexity of encoding. For CUs with complex textures, many factors affect the division of CUs, and it is difficult to set the judgment criteria manually to bring accurate prediction results. Therefore, in this section, we introduce the classifiers selected by the tandem framework and the features used. The proposed algorithm uses random forests as classifiers and utilizes the trained tandem classifier framework to predict the partition pattern of complex CUs, thereby speeding up the coding process and reducing coding complexity.

**Figure 8:**
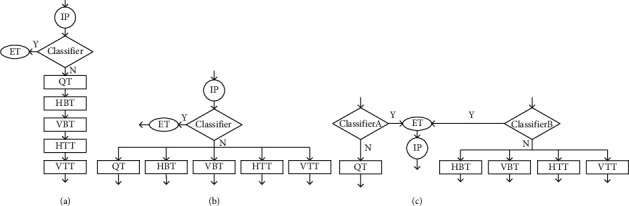
Illustration of 3 partition decision frameworks. (a) Decision tree with early termination of partition. (b) Multi-class selection decision tree. (c) Parallel decision framework.

A classic early termination framework was proposed by Shen et al. [33], as shown in Figure 8(a), where intra prediction (IP) is first performed, and then a classifier is used to determine whether the recursive partition of the coding block is early terminated, if not terminated early, then recursively try all possible partition combinations and select the partition structure with the least rate-distortion cost. In this decision framework, the intra prediction is first checked at each decision layer before the current encoding performs the next division. This checking strategy is not necessary, because when the classifier output is “N,” it will recurse in turn. The 5 division modes will reduce the complexity of the coding process and have limited efficiency. Only when the output of the classifier is “Y,” the division will be terminated in advance, saving coding time. On this basis, Zhang et al. [34] improved the decision tree selection framework, from the recursive selection of CU partition mode to the multi-class selection of CU partition mode, as shown in Figure 8(b). First, perform intra-frame prediction (IP), and then use the classifier to determine whether the CU division is terminated early. If the classifier output is “Y,” the CU division is terminated early; if the classifier output is “N,” in 5 division modes, choose the most likely division method. Although the decision tree framework saves most of the coding time, it will cause the loss of RD performance due to the limited prediction accuracy of the selected partition mode. To improve the prediction accuracy of the selected partition mode and maintain good RD performance, Wang et al. [17] used a parallel decision framework of two classifiers, as shown in Figure 8(c). Classifier A and Classifier B exist independently and are, respectively, used to determine whether the CU performs quad-tree division or multi-type tree division. There are four situations in the parallel selection framework. The first is that when the output of classifier A is “Y” and the output of classifier B is “N,” the quadtree division will be terminated in advance, and then in the four division methods (HBT, VBT, HTT, VTT) select the most likely division method. The second is that when the output of classifier A is “N,” the CU will select the quadtree division mode, and when the output of classifier B is “Y,” the multi-type tree division will be terminated early. The third is that when the choices of classifier A and classifier B are both “Y,” the CU division is terminated early, which saves coding time. The fourth is that both classifier A and classifier B output “Y” at the same time, and the current coding block does not terminate the division in advance. Due to its high flexibility, the parallel selection framework reduces the risk of misprediction, improves RD performance, and reduces coding complexity.

**Figure 9:**
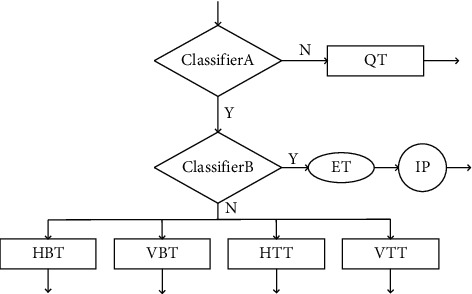
The proposed tandem classifier decision framework.

The classifiers proposed above have more or fewer limitations in reducing the coding complexity. To reduce the coding time as much as possible and speed up the coding process, we propose a tandem classifier framework, as shown in Figure 9. In this structure, two classifiers are used in series and parallel, and there are 3 cases. The first is that when the output of classifier A is “N,” the CU will perform QT division and terminate the multi-type division. The second is that when the output of classifier A is “Y” and the output of classifier B is “N,” the CU skips the QT division, conducts the MTT division test, and selects the partition type with the highest probability. Third, when the selections of classifier A and classifier B are both “Y,” the CU terminates the division early and skips all partition types.

#### 3.2.1. Feature Selection and Analysis

Since VVC/H.266 adopts a complex quadtree nested multi-type tree partition model, the CU partition is particularly sensitive to texture information, so it is very important to select representative features. The new division method of VVC/H.266 can more effectively fit the texture features in the video image and can divide the image with a richer CU block shape and CU block size. Simple texture blocks are always divided very large, while complex texture blocks are divided very small. Therefore, in H.266/VVC intra prediction, the texture of the video content greatly affects the division of the CU. To adapt to this new coding feature and find the features that can determine the type of coding block division, we choose three representative information categories: global sample information, local sample information, and contextual information.

1. *Global Sample Information*. To terminate the coding block division in advance, considering the content complexity of the current CU, we adopt 5 kinds of global samples, including the size of the current coding block (size), variance (var), the ratio of horizontal texture to vertical texture (Gh/Gv), the ratio of the maximum gradient to the maximum number of pixels (Gm/N), and the normalized gradient (Gh+Gv/S). The direction of the CU division is more inclined to the direction of the texture. The gradient in a certain direction reflects the changing trend of pixels in this direction, that is, the gradient can vividly reflect the direction of the texture. The horizontal direction gradient (Gh) and vertical direction are based on the Sobel operator Orientation gradient (Gv) composition.
2. *Local Sample Information*. Due to the new quadtree nested multi-type tree division type, in the area of multi-type tree division, considering the small suburban area of the current encoding, we need local information to represent texture features. For example, the absolute difference (UBD) between the upper half and the lower half of the current coding block, the absolute difference (LRD) between the left half and the lower half of the current coding block, and the complexity difference (SCCD) of the sub-CU. When the texture complexity of the sub-CUs obtained by division is very different, the probability that the current coding block is further divided into smaller coding blocks is very high, so it is represented by the complexity difference of the sub-CUs (SCCD).
3. *Context Information*. Based on the similarity of texture complexity and depth between adjacent CUs, we, therefore, adopt two kinds of context information, including the complexity information (NCC) and depth information (NCD) of adjacent CUs. Considering the complexity information and depth information, acquire the texture complexity and depth values of 5 adjacent CUs in the order of adjacent left, upper left, upper, upper right, and lower left, and calculate the maximum and minimum values, respectively.

**Figure 10:**
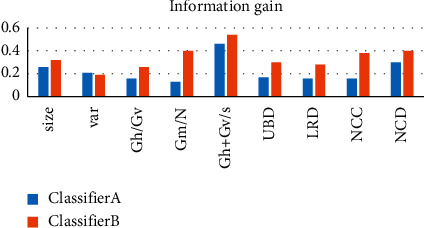
Information gain for selected features.

To demonstrate the effectiveness of feature selection, it is evaluated using Information Gain Attribute Evaluation (IGAE), which uses information gain [35] as a metric for data classification. Therefore, we use the entropy difference between before and after the classification of the dataset to calculate the information gained.  Figure 10 shows the features selected for Classifier A and Classifier B. As can be seen from Figure 10, the features of the two classifiers are consistent, but the information gain values are different.

#### 3.2.2. Model Establishment and Training

Random forest is an ensemble model with a decision tree as the base learner, which is characterized by low variance and low bias and is divided into two parts, namely, random and forest. Compared with the traditional single decision tree, the random forest has the following advantages: First, due to the two randomness, the model has a strong anti-overfitting ability and is relatively stable. Second, the output result is jointly determined by each decision tree, so it overcomes the instability of a single decision tree. Third, it can process high-dimensional data without feature selection, and can also get feature importance ranking. Fourth, provide the class_weight = balanced parameter, which can handle unbalanced data.

We regard the multiple division of CU as a multi-classification problem and use CART as the basic decision tree algorithm of the random forest classifier to build a model to predict the division result. CART is constructed by generating the feature and threshold corresponding to the minimum Gini coefficient at each node. Binary tree. Assuming that there are *k* classes, and the probability that the sample point belongs to the kth class is *p*_*k*_, then the definition formula of the Gini index of the probability distribution is

(2) $$\begin{array}{c} Gini\left(D\right)=\overset{k}{\underset{k=1}{\sum }}p_{k}\left(1-p_{k}\right)=1-\overset{k}{\underset{k=1}{\sum }}p_{k}^{2}\text{ .} \end{array}$$

Among them, *p*_*k*_ represents the probability that the selected sample belongs to the *k* category, and the probability that this sample is wrongly classified is 1 − *p*_*k*_.

In the candidate attribute set A, we select the attribute that minimizes the Gini coefficient after division as the optimal division attribute. If the sample set D is divided into two parts *D*_1_ and *D*_2_ according to whether feature *A* takes a certain possible value a, then under the condition of feature *A*, the definition formula of the Gini coefficient of set *D* is

(3) $$\begin{array}{c} Gini\left(D,A\right)=\frac{\left|D_{1}\right|}{\left|D\right|}Gini\left(D_{1}\right)+\frac{\left|D_{2}\right|}{\left|D\right|}Gini\left(D_{2}\right). \end{array}$$

**Figure 11:**
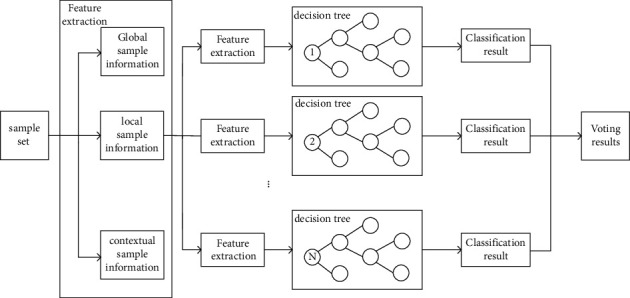
Schematic diagram of the model of the classifier.

Figure 11 shows the offline training process of the classifier. The detailed steps are as follows:

 
*Step 1*. Select 40 frames from each video sequence and encode them with full intra-frame configuration to build the training set of the random forest classifier.
 
*Step 2*. Let the training data set of the node be *D*, and calculate the Gini index of the existing features for this data set. At this time, for each feature A, for each value a that may be taken, divide *d* into two parts *D*_1_ and *D*_2_ according to whether the test of the sample point *A* = *a* is “Yes” or “No,” and use the above formula Gini (*A*) to calculate the Gini coefficient when *A* = *a*.
 
*Step 3*. Among all possible features A and all their possible segmentation points a, select the feature with the smallest Gini coefficient and its corresponding possible segmentation point as the optimal feature and optimal segmentation point. Relying on the optimal feature and the optimal split point, two sub-nodes are generated from the current node, and the training data set is allocated to the two sub-nodes according to the characteristics.
 
*Step 4*. Recursively call Step 1 and Step 2 on the two child nodes, and repeat the above steps until the training of *N* decision trees ends.
 
*Step 5*. After the training of N decision trees is completed, a random forest is formed, and then the classifier is used to classify the current sample. Each tree in the forest independently determines the classification result, and the final classification result takes the same judgment as the prediction of the current sample and obtains CU the best partitioning mode.

Table 1 illustrates the training parameters of the random forest classifier. The training set is derived from some video sequences of the JVET official standard test sequence set, including “RaceHorses,” “BQMall,” “Johnny,” and “ParkScene.”

### 3.3. Overall Algorithm Framework

**Figure 12:**
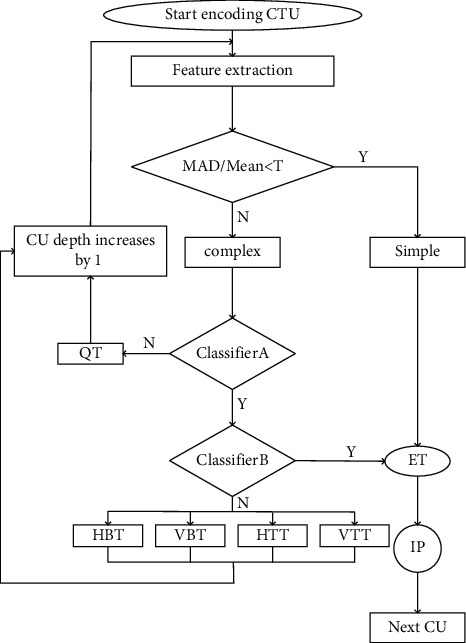
The proposed algorithm flow.

According to the analysis results in the previous section, CUs are classified according to texture complexity, and corresponding acceleration strategies are adopted for different categories of CUs. The fast CU partition decision algorithm framework proposed in this paper is shown in Figure 12.

The specific process is

 
*Step 1*. Calculate all pixel values in the CU and the mean value of the absolute difference of the CU pixels, and then compare the mean value of the absolute difference of the CU pixels with the mean value of the CU pixels. If the given threshold conditions are met, we classify the current CU into two categories: simple and complex.
 
*Step 2.* For simple types of CUs, we terminate the CU partition early, perform intra prediction, and then enter the next CU.
 
*Step 3*. For complex types of CUs, train a tandem classifier decision framework. If both classifiers output “Y,” the CU partitioning process is the same as the simple-type CU.
 
*Step 4.* If the output of the classifier A is “N,” the current CU performs quadtree division, and the divided sub-CUs perform the process again.
 
*Step 5.* If the output of classifier A is “Y” and the output of classifier B is “N,” the current CU performs multi-type tree division, selects the division type with the highest probability, and the divided sub-CU performs the process again.

## 4. Experimental Results and Analysis

Our proposed algorithm is implemented on VVC official test platform VTM-10.0, and the performance of the algorithm is tested. The hardware configuration of the test environment is: processor Intel(R) Core(TM) i5-10500G CPU, 3.20 GHz frequency, 8.0 GB RAM, Windows10 64-bit operating system, and the software development tool is Microsoft Visual Studio 2017. When testing the performance of the algorithm, the standard test video sequences recommended by JVET are encoded in the All Intra (AI) access mode, and the number of encoded frames for each sequence is selected as 50. QP = is set to {22, 27, 32, 37} when encoding. When evaluating the performance of fast video coding algorithms, not only the computational complexity of coding but also coding efficiency and image quality after coding and decoding should be considered. Therefore, the performance of the video encoding algorithm can be measured by two indicators: BDBR and encoding time-saving. Equation (4) is the calculation method for encoding time savings.

(4) $$\begin{array}{c} \Delta T=\frac{T_{vtm}-T_{pro}}{T_{vtm}}\times 100\%\text{,} \end{array}$$

where *T*_pro_ represents the actual encoding time of the proposed algorithm and *T*_*vtm*_represents the time-consuming of the original encoder VTM10.0.

### 4.1. Performance Analysis

Table 2 shows the coding performance of the proposed fast CU partition decision algorithm compared to the VTM10.0 encoder, including RD performance and complexity reduction performance. From Table 1, the following conclusions can be drawn: when the coding structure is full intra-frame coding, the proposed fast CU partitioning algorithm significantly reduces the complexity of CU partitioning, while the coding efficiency decreases slightly. On average, the encoding time was reduced by 52.63%, while BDBR only increased by 1.36%. For different video sequences, the complexity reduction effect of the fast CU partition decision algorithm is similar. The minimum value achieved on the video sequence “RaceHorsesC” is 49.23%, and the maximum value achieved on the video sequence “BasketballDrive” is 60.67%. This shows that the proposed algorithm is stable and can achieve a good complexity reduction effect on different video sequences. In terms of coding efficiency loss, the coding efficiency loss of this paper is the lowest at 0.78 and the highest at 2.83%. Among them, for the test sequence with complex texture, it will cause greater coding efficiency, such as “BasketballDrive” in the B sequence, “BQMall” in the C sequence, etc. For test sequences with simple textures, it will cause less loss of coding efficiency, such as “RaceHores” in D sequences.

**Figure 13:**
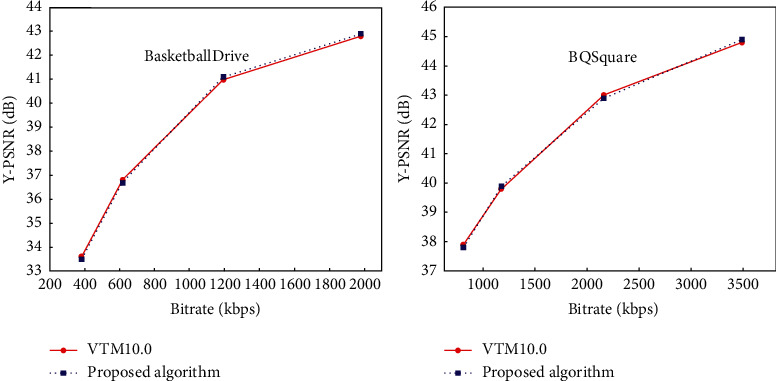
Comparison of the RD performance curve of this algorithm with VTM10.0.

To further estimate the rate-distortion performance of the proposed algorithm, the rate-distortion performance of sequences with different resolutions is counted.  Figure 13 shows the comparison of the RD curves of this algorithm and the standard test model VTM-10.0 for the video sequences BasketballDrive (1920 × 1080) and BQSquare (416 × 240). It can be seen from the figure that the rate-distortion curve of this algorithm coincides with the rate-distortion curve of VTM-10.0, which shows that the BD-rate loss of this algorithm is negligible.

### 4.2. Comparative Analysis

To further evaluate the effect of the proposed algorithm, this article first compares the proposed algorithm with other representative fast algorithms for CU partitioning, including literature [17], literature [26], and literature [31], whose algorithm performance is comparable to that of literature [31]. The performance comparison of the algorithms in this paper is shown in Table 3. Among them, the literature [17] is a QTBT fast decision algorithm based on the joint classifier decision tree structure, which is implemented in the HEVC reference software HM. While literature [26] and literature [31] adopted machine learning–based methods and heuristic methods, respectively, they were implemented in the VVC reference software VTM.

Compared with the algorithm [17], our algorithm reduces coding complexity by 6.95% and saves BDBR by 3.02%, which is a clear improvement. Compared with the traditional algorithm [31], our algorithm decision criterion is adaptively learned by the decision tree and has higher prediction accuracy. Therefore, the algorithm proposed in this article has a lower loss of compression efficiency while keeping the encoding time-saving efficiency the same, and the encoding output bit rate is reduced by 0.14%. Compared with the decision tree–based algorithm [26], the algorithm proposed in this article achieves better results in both RD performance and complexity reduction. Mainly we terminate early for texture-simple coding blocks based on image texture properties. Therefore, compared to the literature [26], the encoding time is saved by about 0.16%, while BDBR drops more by about 0.21%.

### 4.3. Ablation Experiment

**Figure 14:**
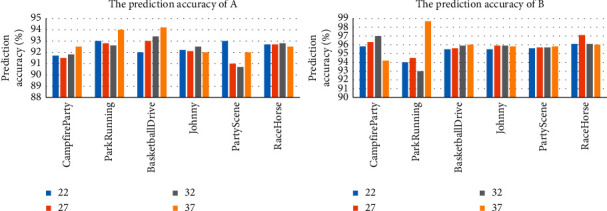
Prediction accuracy of the two classifiers.

In our algorithm, we will conduct an ablation study to analyze the impact of each feature on the overall algorithm. From the experimental results, the features we selected are rich and representative and the two classifiers trained have high prediction accuracy. Therefore, the prediction accuracy of the two classifiers A and B is first calculated.  Figure 14 shows the prediction accuracy of A and B on different video sequences under four QP settings. It can be seen that the prediction accuracy of A is more than 90%. At the same time, for different video sequences and different QP settings, the prediction accuracy is also stable, maintained at 90%–95%. The prediction accuracy of B is more than 93%. Moreover, for different video sequences and different QP settings, the prediction accuracy is also stable, maintained at 93%–97%. Therefore, the proposed VVC intra-coding fast decision algorithm can accurately predict the best CU partition type for complex types of CUs.

## 5. Summary

In this paper, the detailed process of VTM10.0 coding block division is first introduced in detail. Then, the texture complexity is analyzed, and a fast partitioned intra-coding algorithm for CU based on a concatenated decision framework is proposed. The main ideas of the algorithm include: firstly, the relationship between image texture features and CU partition mode is analyzed, and then we design corresponding acceleration strategies for CUs of different texture types, namely, simple types and complex types. The characteristics of the CU partition structure decision are analyzed to terminate the CU partition process in advance. According to the analysis results, three information categories, including global sample information, local sample information, and context information, were selected for CU that did not meet the requirements of early termination of partition, and the classification method with the maximum probability was selected by using the series decision framework designed by us. Finally, we compare our algorithm with other state-of-the-art algorithms, and the proposed algorithm saves 52.63% in encoding time, while BDBR only increases by 1.36%. Although our proposed algorithm significantly reduces the coding complexity, there are still some shortcomings, such as only predicting whether to terminate the partition early or not for simple types of CUs. Combined with the current research results, if the optimal partition mode is directly predicted for all types of CUs, the coding complexity will be further reduced, which is also the direction we can continue to study and improve.

## Figures and Tables

**Table 1:** Training parameters setting.

Parameter name	Parameter settings
Number of feature attributes	10
Number of trees in random forest	10
Tree depth	15
Minimum sample required to split a node	20
Number of categories	5 for classifier A, 2 for classifier B

**Table 2:** Experimental results comparing the proposed algorithm with VTM 10.0.

Sequence type	Test sequence	BD-BR (%)	△*T* (%)
A1(3840 × 2160)	Tango2	1.23	54.25
	Food market4	0.78	55.63
	Campfire	1.18	52.22

A2 (3840 × 2160)	Cat robot1	1.34	53.8
	Daylight road2	1.51	53.62
	Park running3	1.23	51.32

B (1920 × 1080)	Basketball drive	2.83	60.67
	BQ terrace	1.76	55.37
	Cactus	1.57	50.63
	Kimono	1.08	50.18
	Park scene	0.97	49.23

C (832 × 480)	Basketball drill	1.94	52.43
	BQ mall	1.76	50.22
	Party scene	0.91	50.07
	Race horses C	1.08	49.23

D (416 × 240)	Basketball pass	1.43	55.24
	BQSquare	0.86	49.47
	Blowing bubbles	1.03	49.91
	Race horses	0.94	53.17

E (1280 × 720)	Four people	1.62	53.35
	Johnny	1.73	54.31
	Kristen and Sara	1.16	53.48

	Average	1.36	52.63

**Table 3:** Comparison of coding performance between the algorithm in this paper and literature [17], literature [26], and literature [31].

Sequence type	Test sequence	Wang [17]		Yang [26]		Chen [31]		Proposed	
		BDBR (%)	△*T* (%)	BDBR (%)	△*T* (%)	BDBR (%)	△*T* (%)	BDBR (%)	△*T* (%)
B	Basketball drive	4.22	67.98	1.93	59.51	3.09	65.34	2.83	60.67
	BQ terrace	3.47	48.57	2.07	56.07	1.16	49.46	1.76	55.37
	Cactus	4.14	49.20	1.26	51.84	1.74	55.97	1.57	50.63
	Kimono	1.97	65.40	1.9	63.87	1.72	55.31	1.08	50.18
	Park scene	5.08	60.80	1.53	56.6	1.28	56.36	0.97	49.23

C	Basketball drill	6.64	44.61	2.01	48.19	1.91	53.19	1.94	52.43
	BQ mall	4.87	30.19	1.34	47.36	1.79	56.51	1.76	50.22
	Party scene	1.90	40.25	0.6	45.73	0.28	41.76	0.91	50.07
	Race horsesC	2.42	36.24	1.16	48.39	0.84	52.14	1.08	49.23

D	Basketball pass	9.21	49.33	1.28	50.16	2.02	54.15	1.43	55.24
	BQ square	4.53	37.47	0.81	46.06	0.17	32.35	0.86	49.47
	Blowing bubbles	2.36	27.65	0.77	41.56	0.49	43.97	1.03	49.91
	Race horses	2.74	29.33	0.86	43.17	0.54	44.94	0.94	53.17

E	Four people	5.08	43.10	2.75	57.64	2.55	62.27	1.62	53.35
	Johnny	7.33	52.27	3.29	58.98	3.07	62.61	1.73	54.31
	Kristen and Sara	5.15	43.34	2.51	59.19	2.26	60.83	1.16	53.48

	Average	4.44	45.36	1.63	52.15	1.56	52.95	1.42	52.31

## Data Availability

The data used to support the findings of this study are available from the corresponding author upon request.
